# Navigating uncertainties of death: Minimally Invasive Autopsy Technology in global health

**DOI:** 10.1080/17441692.2023.2180065

**Published:** 2023-02-28

**Authors:** Halina Suwalowska, Patricia Kingori, Michael Parker

**Affiliations:** Ethox Centre, Wellcome Centre for Ethics and Humanities, Nuffield Department of Population Health, University of Oxford, Oxford, UK

**Keywords:** Uncertainty, minimally invasive autopsy, minimally invasive tissue sampling, global health, ethics, technology

## Abstract

Global health practitioners and policymakers have become increasingly vocal about the complex challenges of identifying and quantifying the causes of death of the world’s poorest people. To address this cause-of-death uncertainty and to minimise longstanding sensitivities about full autopsies, the Bill and Melinda Gates Foundation have been one of the foremost advocates of minimally invasive autopsy technology (MIA). MIA involves using biopsy needles to collect samples from key organs and body fluids; as such, it is touted as potentially more acceptable and less invasive than a complete autopsy, which requires opening the cadaver. In addition, MIA is considered a good means of collecting accurate bodily samples and can provide the crucial information needed to address cause-of-death uncertainty. In this paper, we employ qualitative data to demonstrate that while MIA technology has been introduced as a solution to the enduring cause-of-death uncertainty, the development and deployment of technologies such as these always constitute interventions in complex social and moral worlds; in this respect, they are both the solutions to and the causes of new kinds of uncertainties. We deconstruct the ways in which those new dimensions of uncertainty operate at different levels in the global health context.

## Introduction

### Establishing the CHAMPS network: ‘solving mysteries to save lives’

**Figure 1.**
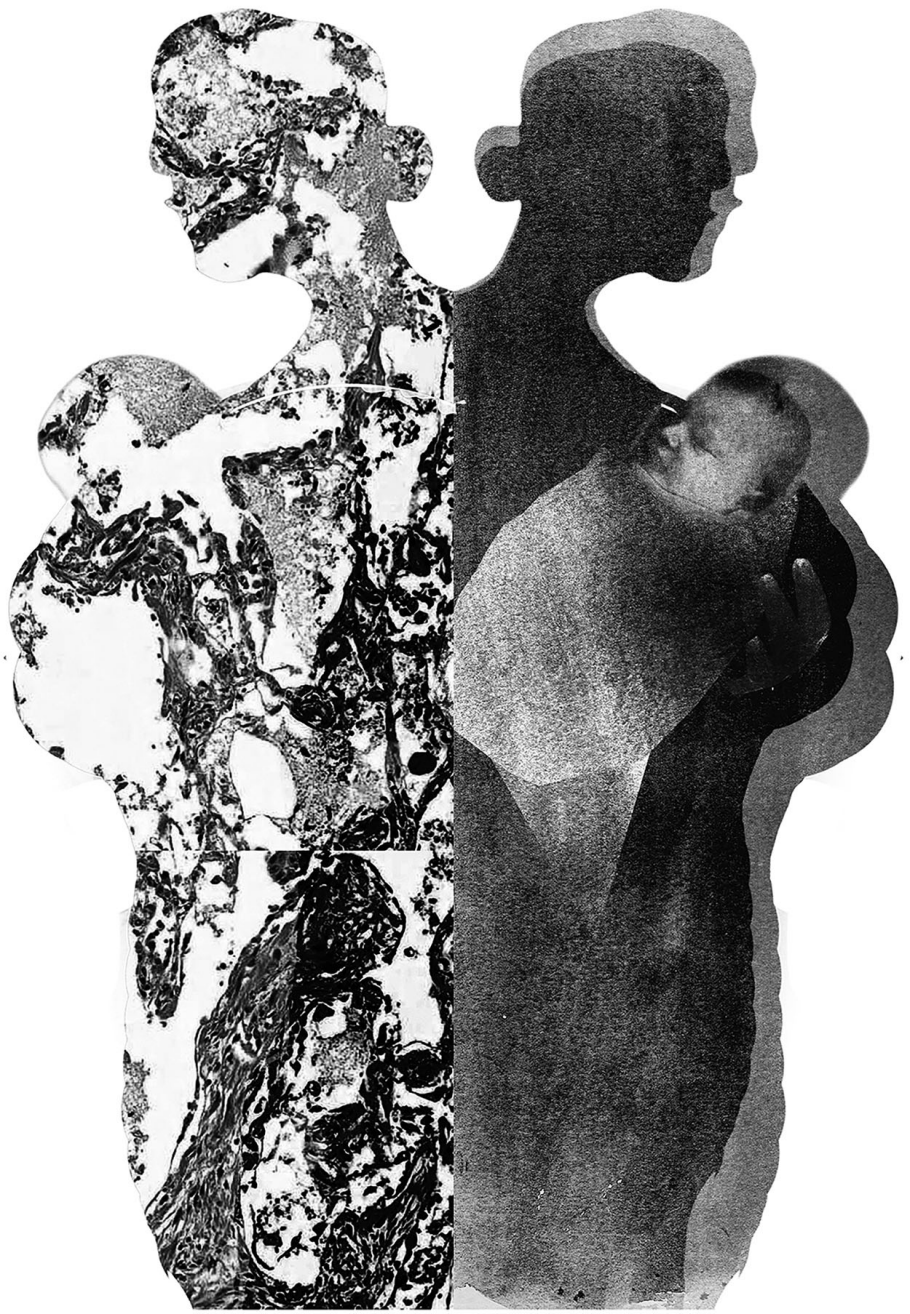
Minimally Invasive Autopsy (MIA), also called Minimally Invasive Tissue Sampling (MITS) is perceived as a technological solution possibly contributing to decreasing mortality in children under five. Copyright © Anna Suwalowska 2022

On 6 May 2015, a press release from the Bill and Melinda Gates Foundation announced the establishment of its Child Health and Mortality Prevention Surveillance (CHAMPS) network. The stated goal of the network was to address the ongoing uncertainty in cause-of-death data for children under the age of five, using a technological solution: minimally invasive autopsy (MIA), also called Minimally Invasive Tissue Sampling (MITS). MIA technology involves using biopsy needles to collect samples from key organs of the body and fluids and so is argued to be potentially more acceptable and less invasive than a full autopsy, which requires opening the cadaver (Castillo et al., 2015; Castillo et al., 2016) (Figure 1).

The CHAMPS initiative was described by Mr Gates as ‘the most exciting, high-stakes work we fund solving mysteries to save lives’ (Gates & Gates, 2017). CHAMPS, a $75-million, 20-year programme, was to be established in areas with high child mortality rates in sub-Saharan Africa and South Asia, with a plan to expand the programme to 20 locations in total as it progressed.

The launch of the programme received considerable publicity. Mr Gates gave media interviews in which he expressed confidence that MIA technology could close an important information gap. The press hailed the MIA technological solution as possibly contributing to decreasing mortality in children: ‘Championed by Bill Gates, innovative autopsies could unlock mystery of early childhood deaths’ (Stiffler, 2017); ‘Bill Gates's Quest to Determine Why Children Are Dying – an interview with Gates about why so many newborns die in developing countries and what he thinks he can do about it’ (Khazan, 2015). This excitement was also shared by other stakeholders. The Centre for Disease Control and Prevention (CDC) Director and CHAMPS collaborator Tom Frieden asserted that it would ‘help us find, stop, and prevent outbreaks’ and ‘not only save children in Africa and Asia but will help to make the world a safer, healthier place for everyone’. Scott Dowell from the Bill and Melinda Gates Foundation hailed the CHAMPS programme as ‘pretty unprecedented’ (Stiffler, 2017).

#### Cause of death uncertainty in global health

CHAMPS’ goal of addressing the uncertainty in cause-of-death data for children relates to a core problem in global health: that of the enduring and interconnected uncertainties in relation to mortality data across all demographics, particularly in the Global South. Death is certain and a biological given; however, at global and national levels, persistent uncertainty surrounds the exact numbers of deaths occurring and the causes of these individual deaths (Byass, 2009; Byass, 2016,). Solving the cause-of-death uncertainty in global health is complex. This knowledge gap is exacerbated by the low measurements of deaths in the Global South due to lack of capacity and a general underdevelopment of health systems. Furthermore, causes of deaths are not collected, as many people die in their homes without having been seen by a qualified medical professional and are buried or cremated with their medical history unknown (Abouzahr et al., 2015; Adair, 2021; Jha, 2014; Setel et al., 2007; Vogel, 2012). A study by Adair (2021) has estimated that 60% of deaths in the Global South occur at home, compared with 27% in high-income countries. Furthermore, there is considerable variation in the percentage of deaths occurring at home; it is highest in countries in South, East and Southeast Asia and sub-Saharan Africa. Adding to this complexity is the fact that even for those who die in hospitals, clinical autopsy – a gold standard for determining the cause of death – is rarely conducted due to low acceptability and lack of facilities and trained pathologists (Anim, 2015; Lishimpi et al., 2001; Mfutso-Bengu, 2002; Tan et al., 2010; Ugiagbe & Osifo, 2012; Wilson et al., 2018). Moreover, diagnostic tools before a death occurs are limited. As a result, cause- of- death assignments on death certification made by physicians are often inaccurate and unreliable.

As a partial solution to addressing the knowledge gap, the World Health Organisation (WHO) has recommended the use of verbal autopsy – a structured interview carried out by a trained professional with relatives or other caregivers to estimate the cause of death when death occurs outside a health facility (WHO, 2016). Verbal autopsy has been utilised in many countries and has contributed to narrowing the information gap; however, it has many limitations. For example, although a verbal autopsy provides an estimation of the patterns of a cause of death at community level, it is not an accurate method for attributing causes of death at the individual level (Byass, 2010). Furthermore, the verbal autopsy tool is not precise when determining the cause of death in children, as symptoms are often indistinguishable (Coldham et al., 2000; Soofi et al., 2015).

With global and national health practitioners and policymakers confronted with the challenge of identifying and quantifying cause of death, the promise of CHAMPS’ MIA technology has engaged a wide range of actors at global, national and local levels who have advocated for MIA technology. This is in addition to stakeholders from the private sector, local research institutes and government agencies all endorsing the promise of MIA technology. To put this in context, incomplete or inaccurate cause-of-death data has been an obstacle faced by several communities; as such, they are further weighed down by the challenge of improving clinical practice and reducing preventable child mortality. According to their aims, CHAMPS’ commitment is consistent with the major global theme of solving malnutrition and poverty and the Sustainable Development Goal 3.2, which aims to eradicate all preventable under-five childhood deaths by 2030 globally (WHO, 2020).

### MIA developments

MIA is an umbrella term that includes a range of technologies used throughout the world with a variety of strategies. In high-income countries, these include the use of MIA imaging techniques to investigate stillbirths and neonatal deaths and to guide needle biopsies (Breeze et al., 2007; Garg et al., 2009; Sebire, 2006; Sebire et al., 2012) and can also be used as an alternative to a coroner's autopsy (Roberts & Traill, 2014). In low-income settings, these technologies remain unrealistic because of the high costs of the technology. In these settings, the MIA procedure has been simplified, with no imaging techniques required. This form of MIA involves using hollow needles to sample key organs of the body and fluids without having to open the body of the deceased (Bassat et al., 2013; Castillo et al., 2015; Cox et al., 2014;).

The first attempt to develop and test the use of MIA for cause-of-death investigation in the Global South was carried out by Castillo et al. (2015) as part of the CaDMIA study. A standardised MIA procedure was developed and tested in a series of 30 autopsies performed at the Maputo Central Hospital, Mozambique. The results, which were published in 2015, concluded that.

*a simplified MIA technique allows obtaining adequate material from body fluids and major organs leading to accurate diagnoses. This procedure could improve the determination of cause of death in developing countries. (*Castillo et al., 2015*, p. 2)*

Further validation studies of MIA have been carried out among different age groups (Bassat, 2017; Bassat et al., 2017; Castillo et al., 2016; Martínez et al., 2016; Menéndez et al., 2017), while a social study has investigated the hypothetical acceptability of MIA across several settings (Maixenchs et al., 2016).

Following the encouraging results of the abovementioned studies, in 2016, the Bill and Melinda Gates Foundation set up the Child Health and Mortality Prevention Surveillance (CHAMPS) network with the aims of providing information on the causes of childhood mortality in the highest-mortality regions of sub-Saharan Africa and south Asia, ending preventable child mortality, and addressing issues around validity and regional representation of data (Farag et al., 2017). The Foundation also established the MITS Surveillance Alliance, which aims to ‘improve cause of death ascertainment through the expansion of the use of MITS globally’ and ‘facilitate the use of MITS by interested institutions and researchers and help grow the network of partners using pathology-based surveillance in various populations, geographies, and contexts’ (MITS Surveillance Alliance, 2022).

### MIA creating new uncertainties

In this paper, we present an analysis of the views of our interviewees on MIA technology in global health, paying particular attention to the MIA technology employed by the CHAMPS programme and the programme itself. Reference to other MIA research is made in the text.

The data shows that while MIA technology was presented by some interviewees as a ‘magic bullet’ solution (Cueto et al., 2013) to the problem of uncertainty about the cause of death in global health, the development and deployment of technologies such as MIA always constitute interventions in complex social and moral worlds; in this respect they are both solutions to and creators of new and enduring uncertainties. Uncertainties created by MIA operate at different levels in the global health context: local, health system and policy levels. First, at local level, uncertainties arise when making requests to carry out MIA and in navigating the complexities of the sociocultural values of communities while meeting the programme's goals. Secondly, at health system level, MIA creates several uncertainties by diverting attention and resources away from more intractable and systemic issues, which remain central in terms of why people experiencing deprivation are being guided towards an unobtainable ‘techno-fix’. Thirdly, at policy level, uncertainty arises in identifying the beneficiaries of the programme and the future ownership of the data collected using MIA technology.

## Methods

The primary research question of this study is: *what are the perceptions of and ethical issues arising from the implementation of MIA technology in the Global South*? Data collection was conducted between January and November 2018.

To answer the above research question, a qualitative approach involving semi-structured in-depth interviews (online and face-to-face) was applied. This is the optimal methodology for answering research questions that shed light on complex, nuanced situations where interpersonal ambiguity and multiple interpretations exist (Austin & Sutton, 2014). In addition, this method allowed us to collect in-depth information by asking clarifying questions and requesting more in-depth information about the reasons for respondents’ initial answers (Blaxter et al., 2006).

A purposive sampling strategy, which means deliberately choosing respondents in order to reflect some features or characteristics of interest (Green & Browne, 2005) was combined with a certain amount of ‘snowball’ sampling. The aim of the chosen sampling method was to explore the views of those involved in the implementation of MIA technology on the global and local level, proponents of other methods, and those who were knowledgeable about mortality measurements in global health. This sampling strategy was best suited to capture a wide range of voices and to map out the range and different types of relevant experiences; it also allowed sampling to be informed by the concurrent data analysis.

Prior to the data collection, a range of organisations, programmes and individuals from the Global South and North were purposively identified. The initial list included CHAMPS network grantees in the USA, Africa and Asia; research funders; researchers who utilised MIA/autopsy in their studies, including the CaDMIA project; pathologists; and global health experts working in organisations invested in collecting or using mortality data, such as the WHO, the World Bank and the Institute for Health Metrics and Evaluation (IHME). As the data collection progressed, the list of interviewees evolved. Further interviewees were identified through snowball sampling – those who were interviewed suggested others who could take part in the study.

47 individuals were interviewed. Interviews were conducted face-to-face where possible, and otherwise via video-calling or by phone, given the diverse geographical spread. The study was approved by the Oxford Tropical Research Ethics Committee (OxTREC Reference: 521-517) as a minimal risk study. Many of the participants were key experts in their field, holding senior positions and significant professional credentials, and described themselves as having several institutional affiliations. The themes explored in the interviews included: current methods of measuring mortality and cause of death in the Global South; developments around MIA implementation; practical ethical challenges; and the future of MIA. The topic guide developed for structuring the interviews was used flexibly, allowing the flow of the interviews to be guided by the responses, without rigid adherence to a structure that might have affected the rapport and the information shared.

Data analysis was an iterative process during the project, conducted in tandem with data collection. The interview data was analysed using thematic analysis, which allowed us to map out the issues, descriptions, interpretations and experiences of the experts interviewed in the study.

Following the first few interviews, a preliminary exploratory analysis was conducted; that is, the data was initially hand-coded on printed documents by labelling fragments of interview extracts with phrases that captured the meaning of the data. The raw data (interview transcripts) was then entered into NVivo, but uncoded. The codes initially generated from the previous round of coding on interviews were entered as nodes. This produced an initial code list. Entering the data into NVivo allowed for further collapsing and merging of codes into initial themes. These were again recoded by going back to the interviews as part of an iterative process to check conceptual validity within codes. During the later recoding steps, there were occasions when codes had to be revisited and re-categorised (Braun & Clarke, 2006). Finally, the codes produced after this round of NVivo recoding were collapsed, resulting in the themes and sub-themes.

The quotations used in this article are available in the online supplement.

## Results

### MIA: a potential solution to cause-of-death uncertainty in global health

The accounts of the MIA stakeholders at the core of the CHAMPS programme highlight their position of certainty that MIA technology will solve the data gap problem in the Global South, thus decreasing global child mortality and ‘saving lives’ (quotation). For these stakeholders, the MIA technology solution in the CHAMPS programme is on course to gather ‘definite’, ‘gold standard’ (quotations), pathology-based information on the causes of stillbirth, neonates and child deaths in the parts of Africa and South Asia with the highest rates of these mortalities. The level of detail and the quality of the information provided by MIA technology was further described as ‘unprecedented’ (quotation) by a CHAMPS stakeholder interviewed for this study. This is because pathology-based surveillance of mortality in children has not previously been carried out anywhere in the world. As the quotation below illustrates, there is a dearth of information about cause of mortality in children:

### Quotation 1 (see supplement 1)

Many interviewees expressed high hopes and expectations for the future outcomes of the CHAMPS programme and the potential impacts of the follow-up interventions. MIA technology is expected, for the first time, to provide countries with ‘real data’ (quotation) about cause of death in children rather than imprecise estimates. Consequently, the technologies and interventions generated based on MIA data are expected to improve national health interventions and decrease mortality in children. This will happen first at CHAMPS locations and then nationally and globally once the data is extrapolated and the programme is rolled out more broadly, according to some interviewees. This would in turn help to lower under-five mortality in all countries where such programmes are carried out. For some interviewees who were not involved in the CHAMPS programme, an ideal outcome would be the ability for countries to build sustainable lab capacity, with a set of people, instruments and tools for surveillance, including mortality surveillance and calibrating verbal autopsy tools. Therefore MIA technology could potentially have enormous leverage on the measurement of population health.

### Ethical and social uncertainties at local levels: MIA technology in the social world

The interviewees generally agreed that MIA technology, by virtue of being less invasive, is more acceptable to the family members of the deceased, both in CHAMPS and in other programmes using MIA. As a result, consent rates for MIA technology in the CHAMPS programme are higher than for full autopsy. As one interviewee put it, ‘the worst – the death of a child – has already happened’, suggesting that the MIA procedure cannot do more harm than the death has already done. However, many interviewees noted that MIA still involves interactions with a recently dead body, which presents several ethical, cultural and logistical challenges; some of these do not differ much from the challenges emerging from a full autopsy procedure, even if MIA is indeed less invasive than full autopsy.

In anticipation of those challenges and to address them, the Social Behavioural Science team at CHAMPS has conducted formative research in order to understand the feasibility of implementing mortality surveillance at each site by, for example, discussing the feasibility of MIA with religious leaders. The socio-behavioural research and continuous community engagement developed and carried out by staff in each country are cases in point. These activities were described by CHAMPS stakeholders as key to the successful implementation of the programme and the long-term commitment from CHAMPS to the study sites. The CHAMPS interviewees explained that the team works to increase the acceptability of CHAMPS at community and individual level. This is done, for example, through community meetings and community sensitisation. As explained by one of the interviewees, the focus of the discussion with communities and families is the protection of the health of children and the possibility of saving their lives, rather than death itself; one interviewee noted that:

### Quotation 2

#### Death and burial practices

The Social Behavioural Science team also addresses the issues relating to CHAMPS being able to carry out their surveillance in such a way as to create minimal disruption to cultural norms and religious practices, priorities and values at the time of a child’s death. As a CHAMPS interviewee put it:

### Quotation 3

As explained by the interviewees, the implementation of MIA technology, whether CHAMPS or others, may collide with cultural and religious obligations towards the dead by interrupting the flow of death rituals and interfering with sacred moments. For example, while MIA is permissible within the Islamic religious tradition under certain circumstances, it interferes with the ritual of washing the dead body and delays the burial, which should take place as soon as possible, usually within 24 h of the death. In the CHAMPS programme, the logistics of handling a dead body for this complex surveillance activity are dictated by time pressure; the MIA procedure needs to be conducted within 12 h after death occurs. The process involves being notified of the death, approaching the family, transporting the body, conducting the MIA procedure, and bringing the body back in time for burial prayers and clothing of the body.

In each site, therefore, local teams have a responsibility to understand the family’s religious and cultural systems before they start the consent process. This is done by involving insiders from the community such as religious leaders, who have been knowledgeable about local issues since before CHAMPS was established. Nevertheless, these interruptions instigate ethical dilemmas and moral uncertainty and may require moral trade-offs. In the quotation below, an interviewee explains the dynamics between the absolute and relative implications and benefits related to MIA, as discussed with Islamic religious leaders:

### Quotation 4

#### Consent process

The interviewees explained that the decision of whether to consent to the MIA procedure is, in most cases, made collectively by the family, even if the consent form is eventually signed by just one person or both parents. In that respect, an MIA request may create a rift in the family when there are divergent opinions on whether or not to consent. Therefore, the complexities relating to MIA requests have been acknowledged and accommodated in the consent process in the CHAMPS programme, as well as in earlier MIA research carried out in other programmes. For instance, families are given ample time to make the decision and all relevant family members are included, even if remotely. As explained by a pathologist from the Global South, in their experience, members of the same community, and even the same family, do not always hold the same views on post-mortem procedures, including MIA, even if they share the same religious or cultural beliefs. As others noted, family decisions are dependent on internal power dynamics, in which age, gender and economic status all play a role. One interviewee noted that MIA technology requests may unintentionally exacerbate gender inequalities, as they put the mother under emotional pressure during the grieving process. Therefore, at some sites a grief counsellor is involved to support family members during the process.

Some interviewees argued that requesting consent for MIA shortly after a death has occurred may compromise the process. This is because grieving families may be so distraught that they may not fully comprehend the information given to them, due to the significant emotional impact of the loss of a child. A suggested solution for this would be for MIA to be part of standard healthcare procedure, with consent not required. However, it has been noted that this may not be a feasible option due to potential distrust of communities; there may be a high level of anxiety among families and communities about ‘mistreatment’ (quotation) of dead bodies, based on a wider mistrust of post-mortem procedures associated with forensic cases or potential misuse of the organs.

#### Ethical uncertainties in offering incentives

One of the ethical issues that was brought up by the CHAMPS stakeholders related to questions around the meaning of death and burial while ensuring that no ethical dilemmas are created for families who consent to MIA. In the interviews, we heard that family members might be put in a ‘double bind’ (quotation) in that they may not want to carry out the procedure because it is against their belief system, but if they do consent to the procedure, they receive a benefit that they would not otherwise have. In one of the locations, for example, the CHAMPS programme offers support for families that consent to MIA, including paying for mortuary bills, transport of the body for burial, and an unconditional financial incentive that will cover the cost of purchasing a coffin or other expenses. For most parents, ascertaining the cause of death of their child was their primary motive for consenting to MIA – as stated at the end of the consent process, it was information they would not have learnt otherwise; however, some – suggested to be the poorest – may have consented because of their poor economic standing. The quotation below illustrates this point:

### Quotation 5

Conversely, one interviewee suggested that knowing the cause of death is not a priority for some families at the time when consent is requested, shortly after the death occurs. At that time, the priority is the burial and taking care of burial-related expenses. Still, it was reported that some parents did not want to know the cause of death when the team contacted them after the MIA process. As interviewees explained, there could be many reasons for this, such as emotional pain after losing a child, perceived lack of benefit in knowing the cause of death, and also already benefiting from the support provided by CHAMPS. In response to such situations, CHAMPS interviewees acknowledged and discussed the complexities of the ethical dilemmas that emerge when approaching families at a time of grieving, the ethical challenges in offering the incentives in the CHAMPS programme, and the way these had been addressed at the time of this study (2018).

### Quotation 6

#### Implications of finding the cause of death

Some interviewees reported that families who consent to MIA benefit by gaining access to the cause-of-death information for their child, which they would not otherwise have. This is because in these settings, diagnostic tools are limited and post-mortem examinations are almost non-existent. However, MIA findings regarding the cause of death, similarly to a full autopsy procedure, may have significant social and psychological consequences for the family members of the deceased. A few interviewees working in the CHAMPS programme have discussed the social and ethical uncertainties that arise for the families after they learn the cause of death. Dealing with complex family and social dynamics after the cause of death is disclosed have been given extra focus so that individuals and families are approached in an appropriate manner. In some cases, for example, interviewees explained that the cause-of-death feedback may trigger a ‘blame game’ (quotation) among the family or cause further distress to the parents if the cause of death turns out to be unknown or a stigmatised disease. A case in point would be disclosing the HIV status of a dead child. While an HIV-positive status does not stigmatise the child who died prematurely, one interviewee explained, it does put a spotlight on the child’s mother, father, and, depending on the individual circumstances, other people. This is important because, as one interviewee suggested, it is likely that the parents of a deceased child who consent to the MIA procedure have not envisaged the consequences of knowing the cause of death, including HIV disclosure, family treatment, and potential family discord. To avoid the stigma associated with HIV and to respect the confidentiality of the findings, the CHAMPS team offers to pay for the parents’ transport to the hospital and report the results there, not in their home, so that the parents are not asked about the child’s cause of death by other family members or neighbours. These complex situations raise many ethical dilemmas for the research team such as how and to whom to disclose the findings; for example, should the father be informed if he is no longer in a relationship with the mother? Should the findings be disclosed to both parents or to the mother first?

### Uncertainties at health system levels

#### Where were you when my child was still alive?

The interviewees also highlighted the uncertainties surrounding the aims of the programme versus their anticipated obligations towards the local communities where the programme is to be carried out for the next 15 years or so. There is a general negative perception in these communities about health systems in terms of availability and quality of services, interviewees reported. A child might be sick for a long time with no outreach from the local health system; however, in contrast, once the child dies, these resources are mobilised to carry out MIA. For parents, who often cannot differentiate between the CHAMPS surveillance programme and the local health system, this is a serious concern, as illustrated by the quotations below:

### Quotation 7

### Quotation 8

With immediate research efforts focusing on dead rather than living children, healthcare issues for the living remain unresolved, with numerous ethical issues emerging as a result. For example, some of the interviewees discussed an ethical concern regarding the responsibilities of the CHAMPS programme towards families beyond identifying and informing them about cause of death. In countries where MIA technology is implemented, healthcare resources are limited; some families who consent to MIA may be living in poverty. Consequently, while some parents might learn the cause of death of their child, in some instances they cannot act on this information, as illustrated by the quotation below:

### Quotation 9

Another interviewee involved in CHAMPS suggested that the programme should consider extending its care to the parents of deceased children. They explained that as grieving the loss of a child is a long-term process, the MIA requests and follow-up process might trigger some negative psychological reactions. Therefore, some suggested that the CHAMPS programme needs to adopt a holistic approach when engaging with mourning families and take responsibility for their wellbeing. There was also a suggestion that CHAMPS should look beyond the medical causes of death and expand in order to address the vulnerability of children dying from unnatural causes of death, such as family violence; this would allow them to be fully embedded in and supportive of the community.

In this light, some interviewees questioned whether the CHAMPS programme could distance itself from the responsibilities involved or whether it should play a greater role in strengthening health systems in the countries where MIA technology is implemented. At the time of this research, CHAMPS was addressing the rift between community expectations and the programme’s activities by identifying activities for supporting maternal and child health that align with the community’s priorities and interests over and above mortality surveillance. For example, in one of the studied country sites, community health fairs were organised in order to provide preventive health information, childhood immunisations and cancer screenings to the community. By doing so, CHAMPS were hoping to gain the community’s trust and demonstrate their desire to improve the health of children and mothers in support and in conjunction with the values of the communities.

#### Local uptake of data, capacity building and sustainability

Many interviewees claimed that MIA technology could potentially provide countries with essential data about cause of death among children for the first time. This will, in turn, enable governments to make ‘smarter choices’ (quotation), target health policies at local and national levels, and allocate resources efficiently. An example of the CHAMPS programme’s benefits to the local community is the ‘data to action’ element, where the findings generated through the programme are developed into local, regional and national action plans for countries. As some interviewees explained, one of the first priorities of ‘data to action’ is that the local communities that provide the context for generating the data are the first to benefit from the action plans developed as an output by CHAMPS. An example given was that finding a cause of death that is a notifiable or reportable disease such as HIV or tuberculosis will generate an immediate public health response, screening the family and potentially members of the community, and then providing support services for them.

Furthermore, CHAMPS members explained that CHAMPS’ findings have the potential to influence local health policies. To do so, the programme works closely with local health institutions and local and national governments in each of the countries they are based, so that government ministers and institutions will understand and contribute to the utilisation of the MIA technology within the country. In some countries, the CHAMPS principal investigators are also employees of the Ministry of Health, which facilitates collaboration. Governments have been part of ‘the DNA’ (quotation) of the CHAMPS programme since the conception and implementation of the project, as illustrated by the quotation below:

### Quotation 10

The abovementioned collaborations have not always been straightforward; some interviewees who were not directly involved in CHAMPS expressed uncertainty about the integration of the data into current health systems. A senior global health specialist not involved in the CHAMPS programme expressed a rather sceptical view, suggesting that involving local governments from a regulatory point of view was ‘trivial’ (quotation) but that the programme was not ‘*all that fascinating to local governments because there is no suggestion that CHAMPS provides a national service*’ (quotation), which indicates that there is ambiguity in the terms of collaboration between the entities. Another interviewee appreciated having ‘real’ (quotation) MIA data on child mortality, as opposed to no data, inaccurate verbal autopsy data or imprecise estimates. However, another criticism is that the number of deaths investigated using MIA will be insignificant when weighed against all deaths occurring in a country. Furthermore, MIA data is not representative of all deaths occurring within a country; it is limited to health facility catchment areas. As such, national governments cannot rely solely on MIA data for decision-making, some claimed, and consequently the future use of the data by governments is not certain. An interviewee involved in the CHAMPS programme also indicated that engaging with the Ministry of Health involves the country’s national politics; therefore, it is sometimes complicated.

The implementation of CHAMPS data has been perceived to have a strong ethical dimension. A global health professional stated that the way this information will be used by communities, and eventually by the countries in whose systems the interventions are carried out, is critical. Pointing out that CHAMPS prioritises the generation of information on the causes of child mortality, they opined that it would be ‘morally reprehensible’ (quotation) if governments were not presented with the opportunity to take advantage of this data.

Similarly, the need for capacity building emerged as one of the compelling themes in the discourse around the obligations of the CHAMPS programme to the countries. Importantly, capacity building has been explained as ‘a great side-benefit of CHAMPS but not the central focus of the programme per se’ (quotation); it is executed if aligned with the focus of the programme on collecting cause-of-death information for children under five, CHAMPS stakeholders explained. Despite this claim, some global health professionals who were not involved in CHAMPS did perceive the programme as potentially capable of playing a critical role in improving countries’ overall health infrastructure. They took the view that CHAMPS could be instrumental in providing the laboratories that countries would need as they moved forward with building their healthcare systems. Some of these activities have already taken place according to CHAMPS members. They listed capacity development training for people working on the CHAMPS system as an example. Essentially, because of CHAMPS, there has been a strengthening of local pathologists and pathology systems for carrying out the laboratory testing and instant pathology that is critical for CHAMPS, as well as further opportunities for local public health leaders to take the information from CHAMPS and act on it.

Moreover, the discussion about capacity building brought up ethical concerns among some of the interviewees, including the responsibility of the CHAMPS programme to ensure that the countries under study become independent in their endeavours. This discussion also brought to light the different expectations of individuals and organisations regarding the delivery and duties of the programme versus the specific goal of identifying cause of death among under-five children.

Another key theme emerging from the data is the sustainability of the CHAMPS MIA technology in the Global South. This technology requires a high financial investment, and the current costs of implementing it are perceived as too high to be covered by national governments, an interviewee explained. Many of the interviewees had doubts that MIA technology as used in the CHAMPS programme would become part of standard healthcare in the Global South – where resources are scarce even for living patients – unless the costs of the technology decrease, the technology is simplified, or it proves economically beneficial for the countries and hence worth investing in. In countries with very limited resources to spend on living patients, justifying the investment of such a substantial amount of money in ascertaining cause of death would be challenging both ethically and politically, as the quotation below affirms:

### Quotation 11

Some accounts that emerged from the interviews suggest ethical concerns around allocating a significant number of resources to a potentially unsustainable technology in low-income settings. One interviewee speculated that even if MIA technology proves to be a good way of diagnosing cause of death, there is no guarantee that this approach will be widely adopted or utilised, even if the results of the CHAMPS study are very positive. It could potentially end up being accepted as a good way of diagnosing causes of death, she/he argued. However, ‘uptake and the results are two very different things’ (quotation) and making MIA a routine method of measuring cause of death would require the advocacy and education of a diverse group of stakeholders in different areas, including medical schools, postgraduate training, and ministries of health*.*

### Uncertainties at policy levels: who benefits and who owns the data

Another uncertainty discussed by the interviewees is associated with the primary beneficiaries of MIA technology. While some of the CHAMPS interviewees argued that the primary beneficiaries of the technology are the communities (even if in the long run) or countries participating in the programme, the predominant view among many interviewees was that the primary audience is the global health community. More specific examples of ‘global beneficiaries’ (quotation) were mostly in the areas of maternal health and childhood health and mortality; they included global funders, researchers, policymakers, global health agencies, the Global Burden of Disease group, and researchers working in the abovementioned medical areas.

Above all, the founder of the CHAMPS network, the Bill and Melinda Gates Foundation, was perceived as the primary beneficiary of MIA technology. The involvement of the Foundation in addressing the uncertainty around cause-of-death data in global health was commented on and disputed by many interviewees involved in global health work and by those working in local health institutions. Some interviewees speculated that the Foundation would use MIA data to make investments and ‘develop new technologies’ (quotation) and that the most likely outcome of MIA technology, and possibly the one most welcomed by the Bill and Melinda Gates Foundation, would be new vaccine technologies. For example, a global health expert pointed out that the Foundation’s emphasis has very much been on children under five, which is ‘coincidentally’ (quotation) the age group where vaccines are important. These speculations were based on the Foundation’s record of investing millions of dollars in technological solutions to global health challenges and the fact that the granularity of the data collected through MIA would allow such developments.

### Quotation 12

Many applauded the Bill and Melinda Gates Foundation’s plans, including the funding of the CHAMPS network, and flagged up their successful track record in global health, highlighting that the Foundation was pushing boundaries in innovation, demonstrating the utility of MIA technology, potentially investing in a field where others were not, and essentially filling a gap. However, others argued that there was no ‘magic bullet’ for mortality reduction and pointed out that the Bill and Melinda Gates Foundation was primarily interested in pursuing high-profile projects, rather than, for example, low-key investment in improving countries’ health systems. It was noted that the Foundation had ‘much deeper pockets than everybody else put together’ (quotation), and with ‘money always wielding power’ (quotation). Therefore, some of those interviewed expressed their concerns about the accountability and transparency of the Bill and Melinda Gates Foundation, as illustrated in the following quotation:

### Quotation 13

#### Future of samples: global or national good?

There was an uncertainty expressed by some interviewees surrounding the future storage and ownership of the biological samples collected using MIA technology. CHAMPS data is open-source (CHAMPS, 2022). However, as explained by an interviewee, the future storage and use of biological materials collected through the MIA technology used by the CHAMPS programme were not clear at the time of conducting the study (2018). The collected specimens are owned by the country and the CHAMPS network. They are held at biobanks in countries with laboratory information systems and are also stored in the USA, ‘mirroring’ (quotation) each other. For every site, there is a Specimen Access Working Group, with a government representative who decides how the site specimens are going to be used. Each country has control over its own specimens and has the right to ask CHAMPS to destroy the samples. There is as yet no agreement or certainty about what will happen to the specimens once the CHAMPS network completes its work in a couple of decades, or who will own the samples in the long term, a CHAMPS interviewee noted. Another interviewee – not a CHAMPS member – speculated that it would be difficult for the governments of the countries participating in the CHAMPS programme to prevent their biological materials from being used once the samples leave the respective countries. In these instances, the national authorities could lose control over the biological materials collected through MIA technology, as explained below:

### Quotation 14

This scenario demonstrates a potential ethical tension over a specimen being perceived as a global good rather than a country-specific material, especially in relation to potential health emergencies such as new diseases that may appear in the future, or other currently unknown scenarios such as the issue of specimen ownership during epidemics.

#### Limitations of ‘magic bullet’ technology.

Full attention has been given to the certainty and standardisation of MIA data; however, paradoxically and despite the expectation that MIA technology will be able to collect ‘definite’ cause-of-death data, our findings suggest that the technology still has some limitations that will not allow it to eradicate uncertainty completely. Some interviewees suggested that while the MIA technology of the CHAMPS programme is accurate in determining causes of death relating to infectious diseases, it is less accurate in recognising non-infectious diseases or, in cases of child death, congenital malformations. Some interviewees argued that childhood mortality is mostly caused by infectious diseases and therefore MIA technology is an appropriate solution; however, others pointed out that the technology might not be useful in addressing the data gap for older age groups. As such, those interviewees argued that MIA technology should not be viewed as sharing the gold standard of full autopsy.

The overwhelming amount of data collected through MIA technology makes it more difficult to interpret the findings and assign a final cause of death, as the quotation below illustrates:

### Quotation 15

Furthermore, the practice of determining ‘the’ cause of death is a complex process, and ‘the’ cause of death might be in fact a ‘whole chain of causations’, leaving the cause of death assignment more ambiguous.

## Discussion

In this paper, we have discussed the different dimensions of uncertainty created by the implementation of MIA technology in the Global South by the CHAMPS programme. The findings show that while MIA technology is perceived by some as a ‘magic bullet’ solution to addressing cause-of-death uncertainty in the Global South, this technology also causes epistemological and ontological uncertainties at local, national and global levels.

This paper contributes to the discourse on the manifestations of uncertainty in global health, particularly the social and ethical uncertainties created by the technological solutions employed in global health interventions. Several aspects of the findings are not limited to research in MIA but are a key point that should be addressed in any research project applying post-mortem procedures or involving low-income populations and different financial sources. For example, the implications of finding the cause of death have similarities to providing a diagnosis for living patients, e.g. reporting genetic test results present in the family or stigmatising a parent who carries the gene (Marsh et al., 2013). The scientific aims of the CHAMPS surveillance to ascertain cause of death in children differ from the expressed immediate needs of the community to improve public health assistance in order to prevent these deaths. This discrepancy of expectations is already known from other research in low-income settings. Similarly, the ethical issues that emerge when offering families an incentive for consenting to MIA raises a critical ethical point relating to local conditions and vulnerabilities, i.e. whether poor families who consent to MIA are invested in knowing the cause of death or are left with an ‘empty choice’ between participating in the study and not finding the cause of death. This is an important finding that resonates with the argument presented by Kingori (2015, p. 764) about the quality of choices offered by research studies carried out in resource-poor settings in the Global South:

[W]hile biomedical research often involves the rhetoric of choice, or the freedom for individuals to choose to participate in research, macro-economic and structural factors frame the options and context in which many prospective participants in Sub-Saharan locations are asked to make such choices […] The discourse of choice creates an illusion of individual freedom and power, without consideration of structural factors which constrain those choices.

The accounts that emerged from this research confirm CHAMPS as a high-profile global health programme and the use of MIA technology as unprecedented. With global and national health practitioners and policymakers confronted with the challenge of identifying and quantifying cause of death, the CHAMPS programme, using MIA technology, may have a significant impact on global health by contributing to a decrease in the mortality of children under five. There is an agreement that technology has played a significant role in reduction of child mortality up until now (Howitt et al., 2012). Notwithstanding, there are also critical voices arguing that the Bill and Melinda Gates Foundation has turned to a narrowly conceived understanding of health ‘as the product of technical interventions divorced from economic, social, and political contexts’ (Birn, 2005; McGoey, 2009). However, many applauded the Foundation’s plans and flagged up their successful track record in global health. Conversely, MIA technology may only partially solve the problem of cause of death in global health. MIA technology needs to be used in combination with other horizontal solutions, in parallel with improving local health systems, and should be tied to a significant rise in living conditions or an improvement in the already widely-used verbal autopsy methodology.

MIA, like other techno-scientific solutions, is a future-oriented endeavour (Borup et al., 2006) and the long-term effects of introducing MIA technology into communities, such as ‘technological surprises’ (Van Asselt & Rotmans, 2002), unexpected consequences, or ‘side effects’ of technologies, are unknown and difficult to predict. MIA is a technological solution created to find the biological cause of death, but its implementation in practical terms intersects society, metaphysics and biomedicine, bringing these parallel dimensions together. Death is universal, and a biological given; it is also local, culturally structured, and discussed in a metaphysical sense (Selin & Rakoff, 2019). As such, there is no certainty about the way the introduction of MIA will, if at all, change values and social fabric in societies where it interferes with the physical, emotional and social body. It is uncertain, for example, whether families will be more willing to donate the bodies of their children or other family members to MIA, if MIA technology is introduced for other age groups, in order to learn about their cause of death.

In the context of MIA technology, biological samples taken from cadavers are secured in biobanks and not traded per se. Fundamentally, MIA technology is an example of the way dead bodies can serve the living and be used in global health. The biological samples collected through MIA could be translated into new knowledge, which in turn may contribute to creating new technologies and interventions for saving lives. It will be important to explore how the processes of commodification of biological materials and knowledge are pursued and to ascertain the nature of the actors involved. Such future research could answer the following questions: Who profits from commercialising the information collected from dead bodies? How will the commercial profit be shared, and with whom? Is the principle of reciprocity being honoured? It will also be important to investigate the access to, and ownership of, the biological samples that are currently held in their respective countries but also have been exported overseas.

Finally, it will be critical to examine the perspectives of national and local governments on the value of MIA technology in their respective countries and communities. Further research is needed in order to investigate how the use of MIA and its outputs is aligned with national health systems for reducing preventable mortality. This will involve investigating the actual uptake of MIA technology and the way potential interventions will be financed. More research is also needed in order to identify the potential beneficiaries of the programme and answer the questions of how the data is shared, who is able to act on it, and how those decisions are made.

## Limitations

Owing to the time constraints of this research project and the potentially different academic orientation of a policy component, relating to the global implementation of MIA technology versus a qualitative study in the communities where MIA is implemented, the decision was made to focus on gathering empirical evidence for the views and experiences of key experts. This might be perceived as a limitation of the study. The community perspectives presented in the findings are derived from interviews with participants based in the Global South and researchers who have conducted MIA research studies in the Global South. The data analysis is based on their own experiences, not those of the members of the communities who were being discussed. However, many of them did live and work in those communities.

## Supplementary Material

Supplemental Material

## References

[CIT0001] Abouzahr, C., De Savigny, D., Mikkelsen, L., Setel, P. W., Lozano, R., Nichols, E., Notzon, F., & Lopez, A. D. (2015). Civil registration and vital statistics: progress in the data revolution for counting and accountability. *The Lancet*, *386*(10001), 1373–1385. 10.1016/S0140-6736(15)60173-8 PMC7753937 25971224

[CIT0002] Adair, T. (2021). Who dies where? Estimating the percentage of deaths that occur at home. *BMJ Global Health*, *6*(9), E006766. 10.1136/bmjgh-2021-006766 PMC8420738 34479953

[CIT0003] Anim, J. (2015). Autopsy practice in Ghana - reflections of a pathologist. *Ghana Medical Journal*, *49*(2), 112–119. 10.4314/gmj.v49i2.9 26339096 PMC4549826

[CIT0004] Austin, Z., & Sutton, J. (2014). Qualitative research: Getting started. *The Canadian Journal of Hospital Pharmacy*, *67*(6), 436–440. 10.4212/cjhp.v67i6.1406 25548401 PMC4275140

[CIT0005] Bassat, Q. (2017). Minimally invasive autopsy: Welcoming a New tool for cause of death investigation in children in resource-constrained countries. *Journal of Tropical Pediatrics*, *63*(4), 249–252. 10.1093/tropej/fmx045 28645210

[CIT0006] Bassat, Q., Castillo, P., Martinez, M. J., Jordao, D., Lovane, L., Hurtado, J. C., Nhampossa, T., Santos Ritchie, P., Bandeira, S., Sambo, C., Chicamba, V., Ismail, M. R., Carrilho, C., Lorenzoni, C., Fernandes, F., Cistero, P., Mayor, A., Cossa, A., Mandomando, I., … Ordi, J. (2017). Validity of a minimally invasive autopsy tool for cause of death determination in pediatric deaths in Mozambique: An observational study. *PLoS Medicine*, *14*(6), e1002317. 10.1371/journal.pmed.1002317 28632739 PMC5478091

[CIT0007] Bassat, Q., Ordi, J., Vila, J., Ismail, M. R., Carrilho, C., Lacerda, M., Munguambe, K., Odhiambo, F., Lell, B., Sow, S., Bhutta, Z. A., Rabinovich, N. R., Alonso, P. L., & Menéndez, C. (2013). Development of a post-mortem procedure to reduce the uncertainty regarding causes of death in developing countries. *The Lancet Global Health*, *1*(3), e125–e126. 10.1016/S2214-109X(13)70037-8 25104253

[CIT0008] Birn, A.-E. (2005). Gates's grandest challenge: transcending technology as public health ideology. *The Lancet*, *366*(9484), 514–519. 10.1016/S0140-6736(05)66479-3 16084261

[CIT0009] Blaxter, L., Hughes, C., & Tight, M. (2006). *How to research*. Open University Press.

[CIT0010] Borup, M., Brown, N., Konrad, K., & Van Lente, H. (2006). The sociology of expectations in science and technology. *Technology Analysis & Strategic Management: The Sociology of Expectations in Science and Technology*, *18*, 285–298. https://doi-org.ezproxy-prd.bodleian.ox.ac.uk/10.1080/09537320600777002

[CIT0011] Braun, V., & Clarke, V. (2006). Using thematic analysis in psychology. *Qualitative Research in Psychology*, *3*(2), 77–101.

[CIT0012] Breeze, A. C. G., Jessop, F. A., Whitehead, A. L., Set, P. A. K., Berman, L., Hackett, G. A., & Lees, C. C. (2007). Feasibility of percutaneous organ biopsy as part of a minimally invasive perinatal autopsy. *Virchows Archiv: An International Journal of Pathology*, *452*(2), 201–207. https://doi-org.ezproxy-prd.bodleian.ox.ac.uk/10.1007/s00428-007-0548-7 18087719 10.1007/s00428-007-0548-7

[CIT0013] Byass, P. (2009). The unequal world of health data. *PLoS Medicine*, *6*(11), e1000155. 10.1371/journal.pmed.1000155 19956675 PMC2777404

[CIT0014] Byass, P. (2010). The imperfect world of global health estimates. *PLoS Medicine*, *7*(11), E1001006. 10.1371/journal.pmed.1001006 21152416 PMC2994666

[CIT0015] Byass, P. (2016). Minimally invasive autopsy: A New paradigm for understanding global health? *PLOS Medicine*, *13*(11), e1002173. 10.1371/journal.pmed.1002173 27875535 PMC5119692

[CIT0016] Castillo, P., Martínez, M. J., Ussene, E., Jordao, D., Lovane, L., Ismail, M. R., Carrilho, C., Lorenzoni, C., Fernandes, F., Bene, R., Palhares, A., Ferreira, L., Lacerda, M., Mandomando, I., Vila, J., Hurtado, J. C., Munguambe, K., Maixenchs, M., Sanz, A., … Ordi, J. (2016). Validity of a minimally invasive autopsy for cause of death determination in adults in Mozambique: An observational study. *PLOS Medicine*, *13*(11), e1002171. 10.1371/journal.pmed.1002171 27875530 PMC5119723

[CIT0017] Castillo, P., Ussene, E., Ismail, M. R., Jordão, D., Lovane, L., Carrilho, C., Lorenzoni, C., Lacerda, M. V. G., Palhares, A., Rodríguez-Carunchio, L., Martínez, M. J., Vila Estapé, J., Bassat Orellana, Q., Menéndez, C., & Ordi I Majà, J. (2015). Pathological Methods Applied to the Investigation of Causes of Death in Developing Countries: Minimally Invasive Autopsy Approach. *PLoS One*, *10*(6), e0132057. 10.1371/journal.pone.0132057 26126191 PMC4488344

[CIT0018] CHAMPS. (2022). Retrieved 1 July 2022. https://champshealth.org/data/

[CIT0019] Coldham, C., Ross, D., Quigley, M., Segura, Z., & Chandramohan, D. (2000). Prospective validation of a standardized questionnaire for estimating childhood mortality and morbidity due to pneumonia and diarrhoea. *Tropical Medicine and International Health*, *5*(2), 134–144. 10.1046/j.1365-3156.2000.00505.x 10747274

[CIT0020] Cox, J. A., Lukande, R. L., Kalungi, S., Van Marck, E., Van De Vijver, K., Kambugu, A., Nelson, A. M., Manabe, Y. C., & Colebunders, R. (2014). Needle autopsy to establish the cause of death in HIV-infected hospitalized adults in Uganda: a comparison to complete autopsy. *JAIDS-Journal of Acquired Immune Deficiency Syndromes*, *67*(2), 169–176. 10.1097/QAI.0000000000000290 25072614

[CIT0021] Cueto, M., Biehl, J., & Petryna, A. (2013). *A return to the magic bullet? Malaria and global health in the twenty-first century. When People Come First*. Princeton University Press.

[CIT0022] Farag, T. H., Koplan, J. P., Breiman, R. F., Madhi, S. A., Heaton, P. M., Mundel, T., Ordi, J., Bassat, Q., Menendez, C., & Dowell, S. F. (2017). Precisely tracking childhood death. *The American Journal of Tropical Medicine and Hygiene*, *97*(1), 3–5. 10.4269/ajtmh.16-0302 PMC5508885 28719334

[CIT0023] Garg, S., Punia, R. P. S., Basu, S., Mohan, H., & Bal, A. (2009). Comparison of needle autopsy with conventional autopsy in neonates. *Fetal and Pediatric Pathology*, *28*(3), 139–150. 10.1080/15513810902772482 19365742

[CIT0024] Gates, B. & Gates, M. (2017). Our 2017 annual letter. Retrieved 1 May 2018. https://www.gatesnotes.com/2017-Annual-Letter?WT.mc_id=02_14_2017_02_OutreachEmail_IN-GFO_&WT.tsrc=INGFO

[CIT0025] Green, J., & Browne, J. (2005). *Principles of social research, Maidenhead*. Open University Press.

[CIT0026] Howitt, P., Darzi, A., Yang, G. Z., Ashrafian, H., Atun, R., Barlow, J., Blakemore, A., Bull, A. M., Car, J., Conteh, L., Cooke, G. S., Ford, N., Gregson, S. A., Kerr, K., King, D., Kulendran, M., Malkin, R. A., Majeed, A., Matlin, S., … Wilson, E. (2012). Technologies for global health. *Lancet (London, England)*, 380. 10.1016/S0140-6736(12)61127-1 22857974

[CIT0027] Jha, P. (2014). Reliable direct measurement of causes of death in low- and middle-income countries. *BMC Medicine*, *12*(1), 19–19. 10.1186/1741-7015-12-19 24495839 PMC3912491

[CIT0028] Khazan, O. (2015, May, 7). Bill Gates's Quest to Determine Why Children Are Dying - an interview with Gates about why so many newborns die in developing countries and what he thinks he can do about it. *The Atlantic* [Online]. Retrieved 1 May 2018. https://www.theatlantic.com/health/archive/2015/05/bill-gates-child-health-mortality-prevention-surveillance-network/392549/

[CIT0029] Kingori, P. (2015). The ‘empty choice’: A sociological examination of choosing medical research participation in resource-limited Sub-Saharan Africa. *Current Sociology*, *63*(5), 763–778. 10.1177/0011392115590093 27182072 PMC4851216

[CIT0030] Lishimpi, K., Chintu, C., Lucas, S., Mudenda, V., Kaluwaji, J., Story, A., Maswahu, D., Bhat, G., Nunn, A. J., & Zumla, A. (2001). Necropsies in African children: consent dilemmas for parents and guardians. *Archives of Disease in Childhood*, *84*(6), 463–467. 10.1136/adc.84.6.463 11369557 PMC1718810

[CIT0031] Maixenchs, M., Anselmo, R., Zielinski-Gutiérrez, E., Odhiambo, F. O., Akello, C., Ondire, M., Zaidi, S. S. H., Soofi, S. B., Bhutta, Z. A., Diarra, K., Djitèye, M., Dembélé, R., Sow, S., Minsoko, P. C. A., Agnandji, S. T., Lell, B., Ismail, M. R., Carrilho, C., Ordi, J., … Munguambe, K. (2016). Willingness to know the cause of death and hypothetical acceptability of the minimally invasive autopsy in Six diverse African and Asian settings: A mixed methods socio-behavioural study. *PLOS Medicine*, *13*(11), e1002172. 10.1371/journal.pmed.1002172 27875532 PMC5119724

[CIT0032] Marsh, V., Kombe, F., Fitzpatrick, R., Williams, T. N., Parker, M., & Molyneux, S. (2013). Consulting communities on feedback of genetic findings in international health research: sharing sickle cell disease and carrier information in coastal Kenya. *BMC Medical Ethics*, *14*(1), 41. 10.1186/1472-6939-14-41 24125465 PMC4016314

[CIT0033] Martínez, M. J., Massora, S., Mandomando, I., Ussene, E., Jordao, D., Lovane, L., Muñoz-Almagro, C., Castillo, P., Mayor, A., Rodriguez, C., Lopez-Villanueva, M., Ismail, M. R., Carrilho, C., Lorenzoni, C., Lacerda, M. V. G., Bassat, Q., Menéndez, C., Ordi, J., & Vila, J. (2016). Infectious cause of death determination using minimally invasive autopsies in developing countries. *Diagnostic Microbiology and Infectious Disease*, *84*(1), 80–86. 10.1016/j.diagmicrobio.2015.10.002 26508103

[CIT0034] McGoey, L. (2009). Philanthrocapitalism and the Greed of Giving: The Adverse Effects of New Philanthropic Players in Health Funding.

[CIT0035] Menéndez, C., Castillo, P., Martínez, M. J., Jordão, D., Lovane, L., Ismail, M. R., Carrilho, C., Lorenzoni, C., Fernandes, F., Nhampossa, T., Hurtado, J. C., Navarro, M., Casas, I., Santos Ritchie, P., & Bandeira, S. (2017). Validity of a minimally invasive autopsy for cause of death determination in stillborn babies and neonates in Mozambique: an observational study. *PLOS Medicine*, *14*(6), e1002318. 10.1371/journal.pmed.1002318 28632735 PMC5478138

[CIT0036] Mfutso-Bengu, J. M. T. T. E. (2002). Ethical jurisdictions in biomedical research. 10.1016/s1471-4922(01)02218-8 11983605

[CIT0037] MITS Surveillance Alliance. (2022). Retrieved 1 July, 2022. https://mitsalliance.org

[CIT0038] Roberts, I. S. D., & Traill, Z. C. (2014). Minimally invasive autopsy employing post-mortem CT and targeted coronary angiography: evaluation of its application to a routine Coronial service. *Histopathology*, *64*(2), 211–217. 10.1111/his.12271 24164418

[CIT0039] Sebire, N. J. (2006). Towards the minimally invasive autopsy? *Ultrasound in Obstetrics & Gynecology*, *28*(7), 865–867. 10.1002/uog.3869 17121416

[CIT0040] Sebire, N. J., Weber, M. A., Thayyil, S., Mushtaq, I., Taylor, A., & Chitty, L. S. (2012). Minimally invasive perinatal autopsies using magnetic resonance imaging and endoscopic postmortem examination (“keyhole autopsy”): feasibility and initial experience. *The Journal of Maternal-Fetal & Neonatal Medicine*, *25*(5), 513–518. 10.3109/14767058.2011.601368 21740313

[CIT0041] Selin, H., & Rakoff, R. M.. (2019). *Death across cultures* (Vol. 9, Science across cultures: The history of non-western science). Springer International Publishing AG.

[CIT0042] Setel, P., Macfarlane, S. B., Szreter, S., Mikkelsen, L., Jha, P., Stout, S., & Abouzahr, C. (2007). A scandal of invisibility: making everyone count by counting everyone. *The Lancet*, *370*(9598), 1569–1577. 10.1016/S0140-6736(07)61307-5 17992727

[CIT0043] Soofi, S. B., Ariff, S., Khan, U., Turab, A., Khan, G. N., Habib, A., Sadiq, K., Suhag, Z., Bhatti, Z., Ahmed, I., Bhal, R., & Bhutta, Z. A. (2015). Diagnostic accuracy of WHO verbal autopsy tool for ascertaining causes of neonatal deaths in the urban setting of Pakistan: a hospital-based prospective study. *BMC Pediatrics*, *15*(1), 144. 10.1186/s12887-015-0450-4 26438252 PMC4595242

[CIT0044] Stiffler, L. (2017, February, 14). Championed by Bill Gates, innovative autopsies could unlock mystery of early childhood deaths. *Geekwire* [Online]. Retrieved 2 July 2019. https://www.geekwire.com/2017/championed-bill-gates-innovative-autopsies-unlock-mystery-early-childhood-deaths/

[CIT0045] Tan, G. C., Hayati, A. R., & Khong, T. Y. (2010). Low perinatal autopsy rate in Malaysia: Time for a change. *Pediatric and Developmental Pathology*, *13*(5), 362–368. https://doi-org.ezproxy-prd.bodleian.ox.ac.uk/10.2350/09-03-0623-OA.1 20367214 10.2350/09-03-0623-OA.1

[CIT0046] Ugiagbe, E. E., & Osifo, O. D. (2012). Postmortem examinations on deceased neonates: A rarely utilized procedure in an African referral center. *Pediatric and Developmental Pathology*, *15*(1), 1–4. 10.2350/10-12-0952-OA.1 21991941

[CIT0047] Van Asselt, M., & Rotmans, J. (2002). Uncertainty in integrated assessment modelling. *Climatic Change*, *54*(1/2), 75–105. 10.1023/A:1015783803445

[CIT0048] Vogel, G. (2012). How Do You count the dead? *Science (American Association for the Advancement of Science)*, *336*(6087), 1372–1374. 10.1126/science.336.6087.1372 22700899

[CIT0049] WHO. (2016). Verbal autopsy standards: the 2016 WHO verbal autopsy instrument. Retrieved 1 May 2018. https://www.who.int/healthinfo/statistics/verbalautopsystandards/en/

[CIT0050] WHO. (2020). Children: improving survival and well-being. Retrieved 30 July 2021. https://www.who.int/news-room/fact-sheets/detail/children-reducing-mortality

[CIT0051] Wilson, M., Fleming, K., Kuti, M. A., Looi, L., Lago, N., & Ru, K. (2018). Access to pathology and laboratory medicine services: a crucial gap. *The Lancet*, *391*(10133), 1927–1938. doi:10.1016/S0140-6736(18)30458-6 29550029

