# Supplementary material for: Navigating uncertainties of death: Minimally Invasive Autopsy Technology in global health
Source: Glob Public Health. 2023 Feb 28;18(1):2180065. doi: 10.1080/17441692.2023.2180065 (PMC9988304; doi:10.1080/17441692.2023.2180065)
Supplement: Supplemental Material [file RGPH_A_2180065_SM2950.docx]

**List of quotations**

*Quotation 1*

*It really was to fill a gap that was troubling to many people, which was when you ask the question ‘What is really killing kids?’, the answer was we really don’t have very good information. The best we have is verbal autopsies and the best we can do with verbal autopsies is to put these deaths into large buckets like diarrhoea, probably pneumonia, but in terms of categories that would allow us to make decisions about what vaccines to invest in, we didn’t have that.*

*Quotation 2*

*People are sensitive about death; death is not a nice thing to talk about. But if you are talking about pregnancy and the birth of a child and seeing a child grow, that’s a happy thing. So that is what we are trying to propagate out there, to say that we are more interested in knowing [about] things that can keep you healthy. When you start talking about that, then they will tell you, ‘But you know we have dump sites here where children are eating. That is why they are sick’, and then you realise they know what is going on.*

*Quotation 3*

*So our biggest challenge that is just part of the project is this issue of the question of the acceptability of the MITS [Minimally Invasive Tissue Sampling] procedure itself and how do we ensure that the cultural belief systems that people employed to make sense of the death of their child are not disregarded, disrespected, minimised or violated by what we do?*

*Quotation 4*

*If people say that ‘My religious tradition says this is not an acceptable procedure’, talking with religious leaders and asking ‘Is that hard and fast, is there any way in which the negotiation of that is more complicated than a simple no’ and if it is not we can’t really go any further, from my point of view, and we haven’t as a site. But within Islam, broadly related to the question of autopsies in general under certain circumstances or related to the MITS procedure specifically, the whole idea within Islam that moral mandates and teachings within the tradition always take place within the kind of framework of assessing benefits in the context of the risk, or the context of the prohibition that if this can be done for the greater good of the community then it can become something acceptable even if it might have been against the teachings in the first place. And that’s not something specific to the question of MITS or the question of autopsy, that’s part of the religious tradition in really wonderful and complicated ways in general.*

*Quotation 5*

*(…) So you find the consent becomes much easier because they are getting transport which is needed. When a child dies and they don’t have the means to ferry the child home then they ask the healthcare workers to allow them to wrap the child and take the child on a public transport vehicle, and people will not know they are carrying a dead child to their rural home in a rural area. So when we come to them and say they will now have transport and there will be a coffin and someone will take care of the mortuary bill, then you will find them consenting.*

*Quotation 6*

*(…) So how do we make sure that we are not being unduly coercive and that we remain neutral in approaching a family (…)? So how do we make sure that our ways of talking with the family aren’t trying to incentivise them in regard to the benefit while minimising the potential risk or the potential of this not being acceptable to them? How do we make sure that we remain neutral, and we present them with all of the information and then help them to make a decision? And how do we make sure that we do that at the time, just after the death of their child, in a way that is sensitive to all those issues, knowing the question of timing is front and centre for us (…)? I think we have done pretty good at this actually, is to put procedures in place to address those issues and to work and to coach and to roleplay with our teams around their consent processes and the family engagement processes so we can look at those issues, but I can’t say that that dilemma will never arise in a family’s life. I don’t think that is possible.*

*Quotation 7*

*They [parents] say, ‘Why are you coming to us when the child is dead? Why didn’t you come to us before the child had died?’ (….) ‘We had trouble with this child when the child was alive, there was no one to help for transport, why are you offering transport now the child is dead?’.*

As much as we try to explain to them that we couldn’t come before, because ours is a mortality survey, for them it is that part that is a problem.

*Quotation 8*

*One of the concerns is that from the families that have not heard about this study before death and then they get to hear about it at the time their child dies, they begin to ask, ‘Why now, why didn’t I see you when my child was ailing, where were you? You are now coming with all these ideas you are trying to explain.’*

*Quotation 9*

*If there are some diagnoses that have been made out of the procedures, and a treatment recommendation is made, it is way out of reach of the families. […] Who takes care of that in terms of finances? And that testing can be very expensive. There could be some hereditary conditions, there could be some congenital disorders. This mother has lost her child or lost her pregnancy due to some problem that will need further treatment. Do you just give her those results and stop there? What would be the use of handing her over to a Ministry of Health system which you know is not going to be of much help anyway?*

*Quotation 10*

*So, all of the CHAMPS sites were at the request of local leadership and local government and it has sort of built up from there. The sites make it a priority to continue to engage and work with local and national government, and public health leaders, on the CHAMPS results, to make sure that CHAMPS is analysing and producing the data that are most important for local government decision-makers, so that feedback should be going on all the time.*

*Quotation 11*

*A lot of countries are spending, let’s say, a maximum of $100 per person per year on total health expenditure, so to spend $1,000 on somebody who has died, just to find out what they have died of, is pretty difficult in policy terms to argue in that sort of scenario. (Global North, Global Health Professional, Epidemiologist)*

*Quotation 12*

*I may be putting words into [Bill] Gates’ mouth. I haven’t had that conversation with him, but I can see from the degree of exactitude that is going into the pathogen identification that it is clearly linked to, maybe not vaccines, but it is clearly linked to some intervention strategy and since it is coming from Gates and that is what Gates does, I am putting two and two together.*

*Quotation 13*

*The difficulty about Gates [Foundation]is, because they are a private institution, they may get to a position that the evidence says it is not so good, but their belief may still override that, and they may choose to keep on doing it. But they may not – it just depends what actually happens.*

*Quotation 14*

*(…) Once a bit of the dead person’s lung is on dry ice in Atlanta, or somewhere like that, then the national authority has effectively lost control and if they decided to keep it and some new disease emerged or some new analytical methods emerged and they suddenly had to do something with those samples in 20 years, it would be very difficult to stop them, I suspect.*

*Quotation 15*

*I think another risk is, it might seem paradoxical but for each death we have so much information, just orders of magnitude more information than we are used to having about possible causes of death and although that is a good thing for assigning the definitive cause, it does raise a whole bunch of challenges when you have multiple positive tests from PTRs and cultures and histopathology and you have to put that all in the mix. So the process that is called Decode Panel where you get the obstetricians and the neonatalologists all together and assign the definitive cause of death and the chain of causes is an area where we continue to need to work and standardisation. Because there is more information than has been available historically, the Decode Panels are breaking new ground all the time.*
